# Clinical Features of Infectious and Autoantibody Encephalitis

**DOI:** 10.15844/pedneurbriefs-29-4-7

**Published:** 2015-04

**Authors:** John J. Millichap, J. Gordon Millichap

**Affiliations:** 1 Division of Neurology, Ann & Robert H. Lurie Children's Hospital of Chicago, Chicago, IL; 2 Departments of Pediatrics and Neurology, Northwestern University Feinberg School of Medicine, Chicago, IL

**Keywords:** MRI, Autoantibody, Encephalitis, Infection, Outcome

## Abstract

Investigators from University of Sydney, Australia; John Radcliffe Hospital, Oxford, UK; Imperial College, London, UK; and other centers, studied infectious, immune-mediated/autoantibody associated and unknown forms of encephalitis, including frequencies, clinical and radiological phenotypes, and long-term outcome.

Investigators from University of Sydney, Australia; John Radcliffe Hospital, Oxford, UK; Imperial College, London, UK; and other centers, studied infectious, immune-mediated/autoantibody associated and unknown forms of encephalitis, including frequencies, clinical and radiological phenotypes, and long-term outcome. In a retrospective single-center cohort of 164 Australian children, an infectious encephalitis occurred in 30%, infection-associated encephalopathy in 8%, immune-mediated/autoantibody-associated encephalitis in 34%, and unknown encephalitis in 28%. An etiology was proposed in 118 (72%) patients, of whom 64 (39%) were confirmed, 29 (18%) probable, and 25 (15%) possible. In order of decreasing frequency, subgroups included acute disseminated encephalomyelitis (21%), enterovirus (12%), *Mycoplasma pneumoniae* (7%), N-methyl-D-aspartate receptor antibody (6%), herpes simplex virus (5%), and voltage-gated potassium channel complex antibody (4%). Movement disorders, psychiatric symptoms, agitation, speech dysfunction, CSF oligoclonal bands, MRI limbic encephalitis, and clinical relapse were more common in patients with autoantibodies. Age at presentation was a mean of 5.5 years (range 5 weeks-15.1 years), and 94 (57%) were male. The majority (35%) presented during the Australian winter season. Clinical features were fever (77%), seizures (54%), headache (43%), weakness (37%), agitation (34%), CSF pleocytosis (71%). EEG slowing occurred in 85%, and epileptiform discharges in 21%. Outcome was abnormal in 49% patients, after a median follow-up of 5.8 years. Herpes simplex virus and unknown forms had the worst outcomes. An abnormal outcome was more common in patients with status epilepticus, MR diffusion restriction, and ICU admission. Recognition and treatment of the relatively common immune-mediated/autoantibody-associated cases of encephalitis should be a clinical priority. [1]

COMMENTARY. In this study, using the Granerod classification [2], encephalitis was defined as an acute encephalopathy with >-2 of the following: fever >38 °C, seizures or focal neurologic signs, CSF pleocytosis (>5wbc/uL) or elevated CSF neopterin (>30nmol/L), and EEG slowing or abnormal MRI. Confirmed diagnosis had the organism or autoantibody detected in CSF or brain. A probable diagnosis had serological evidence of acute infection or autoantibody, and a possible diagnosis was based on detection of the organism from stool or nasopharynx. The term infection-associated encephalopathy rather than encephalitis was used for encephalitis related to influenza virus or rotavirus. Encephalopathy is defined as an altered or reduced level of consciousness and change in personality or behavior or confusion lasting >24 hours [3].

In the UK, the Aetiology of Encephalitis Study Group [4] made a diagnosis by first-line laboratory testing in 111 (55%) of 203 patients. HSV was detected in 19%, varicella-zoster virus in 5%, and *Mycobacterium tuberculosis* was identified in 5%. Sixteen (8%) samples showed either N-methyl-d-aspartate receptor or voltage-gated potassium channel complex antibodies. An etiological diagnosis was not determined in one-third of cases, a similar frequency to that found in Australia.
